# Latency following preterm prelabor rupture of membranes before 34 weeks of gestation and its association with perinatal outcomes: a retrospective cohort study

**DOI:** 10.1007/s00404-026-08463-7

**Published:** 2026-05-16

**Authors:** Pilar López-Martínez, Javier Sánchez-Romero, Rosa Muñoz-Porcel, Zoraya Mokachir-Mohsenin, José Eliseo Blanco-Carnero, Paz Crespo-Bañón, Romina Sol Liandro, Laura Hernández-Hernández, Themistoklis Dagklis, Aníbal Nieto-Díaz, Catalina de Paco-Matallana

**Affiliations:** 1 Department of Obstetrics and Gynecology, ‘Virgen de la Arrixaca’ University Hospital, Crtra. Cartagena-Madrid s/n, El Palmar, 30120 Murcia, Spain; 2 Department of Obstetrics and Gynecology, Pediatrics and Surgery, University of Murcia, Murcia, Spain; 3 Maternal-Fetal Medicine, Reproduction and Gynecology Research Group, Biomedical Research Institute of Murcia Pascual Parrilla–IMIB, 30120 Murcia, Spain; 4 Spanish network in maternal neonatal child and developmental health research (RICORS-SAMID, RD24/0013/0018), Instituto de Salud Carlos III, 28040 Madrid, Spain; 5 3rd Department of Obstetrics and Gynecology, School of Medicine, Faculty of Health Sciences, Aristotle University of Thessaloniki, Agiou Dimitriou, 54124 Thessaloniki, Greece

**Keywords:** PPROM, Preterm prelabor rupture of membranes, Neonatal outcomes, Pregnancy adverse outcomes, Prematurity

## Abstract

**Purpose:**

To evaluate the association between latency duration and perinatal outcomes in pregnancies complicated by preterm prelabor rupture of membranes (PPROM) before 34 weeks of gestation, and to assess the role of gestational age at PPROM and amniotic fluid volume.

**Methods:**

This retrospective cohort study included pregnancies complicated by PPROM before 34 weeks of gestation managed expectantly at a tertiary care center. Latency duration was defined as the interval from membrane rupture to delivery. Composite neonatal and pregnancy adverse outcomes were analyzed using latency-based time-to-event methods. Cox proportional hazards models were used to assess the independent associations of gestational age at PPROM and amniotic fluid pocket with adverse pregnancy and neonatal outcomes.

**Results:**

A total of 278 pregnancies were included in the final analysis. Latency duration was strongly associated with gestational age at PPROM, with progressively shorter latency observed at more advanced gestational ages. Larger residual amniotic fluid pockets were independently associated with prolonged latency. In latency-based Cox models, gestational age at PPROM was significantly associated with both pregnancy-adverse and neonatal composite outcomes. In contrast, latency duration itself and residual amniotic fluid volume were not independently associated with adverse neonatal outcomes after accounting for gestational age variables.

**Conclusions:**

In pregnancies complicated by PPROM before 34 weeks of gestation, gestational age at membrane rupture is the principal determinant of latency duration and perinatal outcomes. Latency should be interpreted as a gestational-age-dependent phenomenon rather than an independent predictor of neonatal risk, supporting individualized expectant management that prioritizes gestational age advancement while balancing maternal risk.

**Supplementary Information:**

The online version contains supplementary material available at 10.1007/s00404-026-08463-7.

## Introduction

Preterm prelabor rupture of membranes (PPROM) complicates approximately 2–3% of all pregnancies and accounts for nearly one-third of preterm births [1]. It represents a major challenge in obstetric care, as clinicians must balance the risks associated with prematurity against those related to prolonged expectant management, including intra-amniotic infection, placental abruption, and fetal compromise [1]. Perinatal morbidity and mortality following PPROM are primarily influenced by gestational age at membrane rupture, gestational age at delivery, and the interval between these events, commonly referred to as the latency period [1–3].

Expectant management for patients with PPROM before 34 weeks of gestation in the absence of maternal or fetal contraindications is associated with lower rates of neonatal respiratory distress syndrome, reduced need for mechanical ventilation, fewer neonatal intensive care unit admissions, and decreased neonatal mortality, without a significant increase in neonatal sepsis [1, 4]. These findings have established expectant management as the standard of care in PPROM before 34 weeks, with the primary objective of prolonging pregnancy to allow fetal maturation and completion of antenatal interventions, such as corticosteroid administration and latency antibiotics [1, 4].

The latency period, defined as the interval between membrane rupture and delivery, is a key but highly variable feature of PPROM. Approximately half of patients deliver within the first week after rupture, although latency duration is strongly influenced by gestational age at presentation, with longer latency observed at earlier gestational ages [1]. Reported latency durations vary widely across studies, reflecting differences in patient populations, gestational age at membrane rupture, and management strategies [5]. This variability underscores the clinical relevance of latency as a potential determinant of perinatal outcomes and a target for individualized management [2].

The association between latency duration and perinatal outcomes in PPROM remains controversial. Several studies have reported that, after adjustment for gestational age at delivery, prolonged latency is not independently associated with adverse neonatal outcomes, suggesting that gestational age at birth is the primary driver of neonatal prognosis [3]. Conversely, other reports have suggested that latency duration may exert an independent effect on selected neonatal outcomes in specific populations, including multiple gestations [6]. These seemingly conflicting findings highlight ongoing uncertainty regarding whether latency duration itself influences neonatal outcomes beyond its effect on gestational age at delivery [7].

Maternal complications are a central component of the risk–benefit balance of prolonged latency following PPROM [1]. Clinically apparent intra-amniotic infection and postpartum infectious morbidity remain common, particularly at earlier gestational ages, and several studies have reported an increased risk of chorioamnionitis and placental abruption with longer latency durations [8, 9]. However, these maternal complications have not consistently translated into worse neonatal outcomes when pregnancies are closely monitored and timely delivery is undertaken [10]. Importantly, much of the existing literature is limited by heterogeneous gestational age ranges, inclusion of periviable pregnancies with distinct pathophysiology, and analytical approaches that adjust for gestational age at delivery—a key mediator of neonatal outcomes—thereby complicating the interpretation of the independent effect of latency duration [11, 12].

The aim of this study was to evaluate the association between latency duration and neonatal and pregnancy adverse outcomes in a contemporary cohort of pregnancies complicated by PPROM before 34 weeks of gestation managed expectantly. Specifically, we sought to assess whether latency duration was independently associated with adverse perinatal outcomes after accounting for gestational age at membrane rupture and relevant clinical factors, and to explore whether clinically meaningful latency thresholds or subgroups could be identified to inform individualized counseling and management strategies.

## Materials and methods

This single-center study was conducted at Hospital Clínico Universitario “Virgen de la Arrixaca” in Murcia (Spain) and included pregnancies complicated by PPROM before 34 weeks of gestation managed between January 2009 and December 2023. All eligible pregnancies were considered for inclusion in the analysis. The study was conducted in accordance with the principles of the Declaration of Helsinki. Ethical approval was obtained from the local institutional review board (2020-5-8-HCVUA). Informed consent was waived due to the retrospective nature of the study.

### Participants

Eligible for inclusion were singleton pregnancies complicated with PPROM before 34 week gestation with known delivery and neonatal outcome. Pregnancies were excluded if they were affected by structural anomaly, chromosomal abnormality or PPROM before 16 week gestation. Gestational age was determined by the measurement of crown-rump length (CRL) at 11–13 weeks [13]. We searched our computer database (ViewPoint, Webling, Germany) to obtain maternal obstetric characteristics, and outcomes were recorded from labor ward and hospital notes.

Our center adheres to the NICE and local practice guidelines on the management of PPROM [14, 15]. Termination of pregnancy was offered to every pregnancy that suffered PPROM before 23 week gestation. When PPROM occurred after 23 weeks of gestation or when termination was rejected, the patient was offered to admit in obstetrical care admission unit or to follow up with ambulatory care. Intravenous ampicillin 1 gram every 6 h and gentamicin 80 mg every 8 h and oral azithromycin 1 gram every 72 h were administered for 1 week. Maternal signs, fetal ultrasound, cardiotocography and blood tests investigating chorioamnionitis signs (maternal fever, maternal tachycardia, uterine contractions, smelling leukorrhea, fetal tachycardia or leukocytosis) were routinely performed twice a week.

Expectant management was offered to every pregnant woman in the absence of chorioamnionitis signs. Delivery was targeted at least at 34–36 week gestation or even it was individualized at term in some cases. Betamethasone 12 mg was intramuscularly administered for 2 days for lung maturation if delivery was suspected to occur before 34+6 week gestation. Magnesium sulfate, a 4-g bolus and 1 g per hour, was intravenously administered for neuroprotection if delivery was suspected to occur before 32 week gestation.

### Outcome definitions

The primary outcome was composite adverse pregnancy outcome and neonatal composite adverse outcome. Composite adverse pregnancy outcome was defined as stillbirth or medically indicated preterm delivery before 34 weeks of gestation.

Specific antepartum deterioration events (such as intra-amniotic infection, placental abruption, or fetal compromise) could not be consistently or reliably ascertained. Therefore, medically indicated delivery before 34 weeks was used as a pragmatic surrogate of clinically significant maternal–fetal deterioration leading to interruption of pregnancy before the target gestational age. Indications for delivery followed institutional protocols and included suspected infection, non-reassuring fetal status, or other maternal or fetal complications.

In our institution, expectant management after PPROM is generally pursued until 34–36 weeks in the absence of maternal or fetal contraindications. Consequently, delivery before 34 weeks typically reflects clinically relevant worsening rather than elective timing. The individual components of the composite outcome (stillbirth and medically indicated preterm delivery <34 weeks) were also analyzed and reported separately. In addition, a sensitivity analysis was performed using stillbirth alone as the outcome.

The neonatal composite adverse outcome included the need for CPAP or invasive mechanical ventilation due to respiratory distress syndrome, grade III–IV intraventricular hemorrhage, necrotizing enterocolitis, sepsis, retinopathy of prematurity, or anemia requiring blood transfusion.

### Statistical analysis

Gestational age at PPROM was categorized into four clinically relevant groups: <20 week, 20–25 week, 25–30 week, and 30–34 week gestation. Cases with missing outcome data were excluded from the analysis. The primary time-to-event variable was the latency period, defined as the number of days from PPROM to delivery. Latency was additionally analyzed as a categorical variable, categorized as prolonged (>14 days) vs not prolonged (≤14 days).

Continuous variables were assessed for distributional characteristics using the Kolmogorov–Smirnov test, summarized as mean and standard deviation and compared using the Student’s *t* test. Given the sample size, parametric tests were used. Distributional assumptions were assessed, and results were consistent in sensitivity analyses using non-parametric methods. Categorical variables were summarized as counts and percentages and compared using the chi-square test, as appropriate.

Latency duration was described using Kaplan–Meier estimates. Median latency times and interquartile ranges (IQR) were calculated for each gestational age group and graphically represented using point estimates with horizontal confidence intervals.

Potential sources of bias include selection bias due to pregnancy termination in very early PPROM, and confounding by gestational age, which is strongly associated with both latency duration and perinatal outcomes. These were addressed through multivariable adjustment and sensitivity analyses.

Multivariable Cox regression models were constructed to evaluate the association between latency time and composite pregnancy adverse and neonatal outcomes. Variables included in the models were those identified as clinically relevant or statistically significant in univariable analyses. These models should not be interpreted as formal time-to-event analyses for neonatal outcomes, but rather as exploratory analyses assessing whether latency duration differed according to subsequent neonatal status. Multivariable models were fitted using complete-case analysis; therefore, the number of events included in regression models may differ from the crude event counts reported in descriptive tables. Model assumptions were assessed using standard diagnostic procedures, including visual inspection of residuals and assessment of model fit. Additional analyses were performed for the individual components of the composite outcome and for stillbirth alone as a sensitivity analysis. Sensitivity analyses were performed excluding pregnancies with PPROM before 23 weeks of gestation and restricting the analysis to pregnancies before 28 weeks.

Time-to-event analyses used latency from PPROM to delivery as the underlying time scale, with time zero defined at membrane rupture. For pregnancy outcomes, events occurred during the latency period and pregnancies were censored at delivery if no event occurred. For neonatal outcomes, which occur after delivery, Cox models were used to explore differences in latency according to subsequent neonatal status rather than as formal time-to-event analyses. Given that most neonatal events occurred within the first 24–48 h after birth, the temporal separation between delivery and outcome was limited, supporting the clinical interpretability of this approach, although it should be interpreted with caution.

A *p* value < 0.05 was considered statistically significant. All statistical analyses were performed using R statistical software (version 4.3.0, R Core Team, 2023) and Stata/BE 19.5 (StataCorp, College Station, Texas, USA).

## Results

**Fig. 1:**
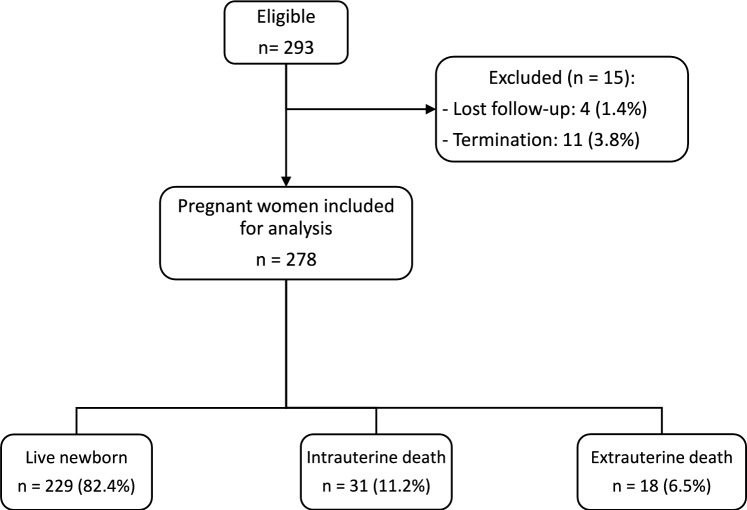
Flow diagram of the study population

During the study period, 293 pregnancies complicated by PPROM before 34 weeks of gestation met the inclusion criteria (Fig. 1). Pregnancy termination occurred in 11 cases, and 4 pregnancies were lost to follow-up. All cases of pregnancy termination occurred in pregnancies with PPROM before 24 weeks of gestation. The 278 remaining pregnancies continued with expectant management and were included in the latency analysis: 229 (82.4%) resulted in a live birth, whereas 31 (11.2%) ended in intrauterine death and 18 (6.5%) ended in neonatal death.

### Baseline characteristics

Baseline maternal characteristics were broadly similar across latency groups (Table 1). Maternal age, body mass index (BMI), parity, and mode of conception did not differ significantly according to latency duration. However, gestational age at PPROM was significantly lower among pregnancies with prolonged latency (29.2 vs 25.0, *p*<0.001). Differences were observed in smoking status and pregestational diabetes.

**Table 1:** Baseline characteristics

	Latency ≤ 14 days (*n*=134)	Latency > 14 days (*n*=144)	Total (*n*=278)	*p*
Maternal age, years	32.0 (6.6)	33.0 (6.4)	32.5 (6.5)	0.231
Weight, Kg	69.1 (13.6)	68.8 (17.8)	68.9 (15.9)	0.916
Height, cm	162.6 (6.4)	163.1 (7.2)	162.9 (6.8)	0.654
BMI, Kg/m^2^	26.3 (5.0)	26.2 (6.6)	26.2 (5.9)	0.947
Mode of conception				
Spontaneous	107/120 (89.2%)	119/133 (89.5%)	226/253 (89.3%)	0.937
ART	13/120 (10.8%)	14/133 (10.5%)	27/253 (10.7%)	
PTB history	13/126 (10.3%)	18/138 (13.0%)	31/264 (11.7%)	0.492
Chronic hypertension	2/125 (1.6%)	1/142 (0.7%)	3/267 (1.1%)	0.521
Diabetes mellitus				
Pregestational DM	4/123 (3.2%)	13/135 (9.6%)	17/258 (6.5%)	0.035
GDM	5/123 (4.0%)	13/135 (9.6%)	18/258 (6.9%)	0.071
Nulliparous	60/130 (46.2%)	62/143 (43.4%)	122/273 (44.7%)	0.642
Smoke	26/134 (19.4%)	15/144 (10.4%)	41/278 (14.7%)	0.035
AF pocket, mm	19.9 (15.9)	21.1 (16.7)	20.5 (16.3)	0.575
AF pocket <20 mm	70/125 (56.0%)	70/138 (50.7%)	140/263 (53.2%)	0.392
Gestational age at PPROM, weeks	29.2 (4.3)	25.0 (5.3)	27.0 (5.3)	<0.001
<20 weeks	7/134 (5.2%)	31/144 (21.5%)	38/278 (13.7%)	<0.001
20–25 weeks	16/134 (11.9%)	39/144 (27.1%)	55/278 (19.8%)	
25–30 weeks	37/134 (27.6%)	42/144 (29.2%)	79/278 (28.4%)	
30–34 weeks	74/134 (55.2%)	32/144 (22.2%)	106/278 (38.1%)	
Time from PROM to delivery, days	7.4 (3.8)	45.6 (37.2)	27.2 (33.0)	<0.001
ACS	120/126 (95.2%)	122/136 (89.7%)	242/262 (92.4%)	0.092
Gestational age at ACS, weeks	29.9 (3.3)	27.4 (3.3)	28.6 (3.5)	<0.001
Gestational age at delivery, weeks	29.8 (4.6)	31.2 (4.5)	30.5 (4.6)	0.011
<24 weeks	16/134 (11.9%)	15/144 (10.4%)	31/278 (11.2%)	0.016
24–28 weeks	27/134 (20.2%)	25/144 (17.4%)	52/278 (18.7%)	
28–32 weeks	40/134 (29.9%)	36/144 (25.0%)	76/278 (27.3%)	
32–37 weeks	51/134 (38.1%)	56/144 (38.9%)	107/278 (38.5%)	
>37 weeks	0/134 (0%)	12/144 (8.3%)	12/278 (4.3%)	
Labour onset				
Spontaneous	70/128 (54.7%)	76/136 (55.9%)	146/264 (55.3%)	0.760
Induced	33/128 (25.8%)	38/136 (27.9%)	71/264 (26.9%)	
No labour onset	25/128 (19.5%)	22/136 (16.2%)	47/264 (17.8%)	
Delivery				
Spontaneous	80/127 (63.0%)	73/139 (52.5%)	153/266 (57.5%)	0.084
Cesarean section	47/127 (37.0%)	66/139 (47.5%)	113/266 (42.5%)	

Continuous variables are summarized as mean (standard deviation) and categorical variables as count (percentage)

Denominators (n/N) are reported for each variable; differences are due to missing data

*BMI* Body mass index, *ART* Assisted reproductive technique, *PTB* Preterm birth, *DM* Diabetes mellitus, *AF* Amniotic fluid, *PPROM* Preterm prelabor rupture of membranes, *ACS* Antenatal corticosteroids

### Perinatal outcomes according to latency duration

**Fig. 2:**
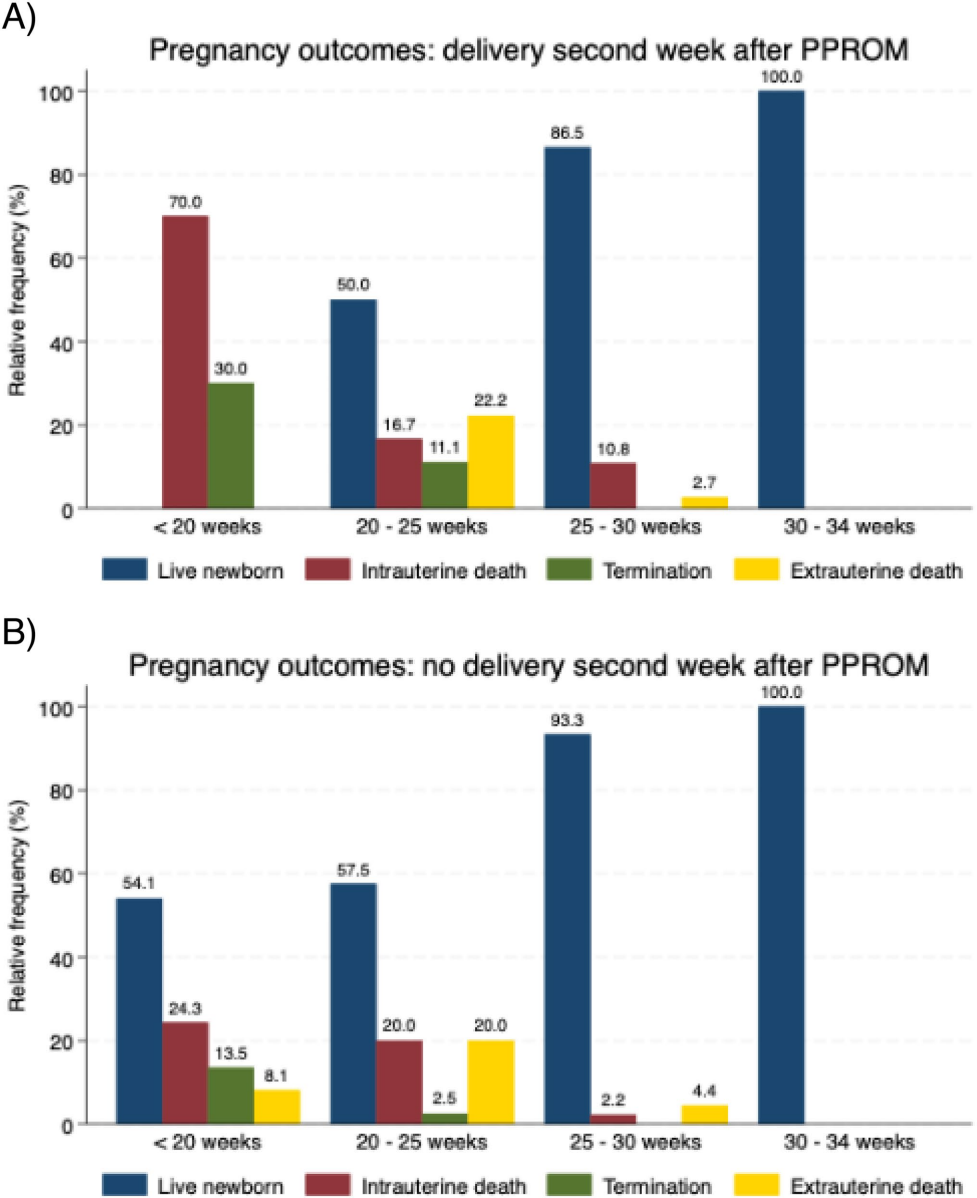
Distribution of neonatal and pregnancy adverse outcomes according to latency duration

No significant differences were observed in overall intrauterine survival between latency groups (*p*=0.174). However, the composite adverse pregnancy outcome occurred in 188/278 (67.6%) pregnancies, including 31 (11.2%) stillbirths and 157 (56.5%) medically indicated preterm deliveries before 34 weeks. The composite adverse pregnancy outcome differed significantly according to latency duration (Table 2 and Fig. 2). Pregnancies with prolonged latency had a significantly earlier gestational age at PPROM, but achieved a later gestational age at delivery and a higher birthweight.

**Table 2:** Perinatal outcomes

	Latency ≤14 days (*n*=134)	Latency >14 days (*n*=144)	Total (*n*=278)	*p*
Latency from PPROM to delivery, days	7.4 (3.8)	45.6 (37.2)	27.2 (33.0)	<0.001
Outcome				
Live newborn	115/134 (85.8%)	114/144 (79.2%)	229/278 (82.4%)	0.174
Intrauterine death	14/134 (10.4%)	17/144 (11.8%)	31/278 (11.2%)	
Extrauterine death	5/134 (3.7%)	13/144 (9.0%)	18/278 (6.5%)	
Birthweight, g	1686.6 (559.8)	1909.9 (730.2)	1800.5 (660.5)	0.008
Apgar score at first minute	7.8 (1.8)	7.4 (2.3)	7.6 (2.1)	0.160
Apgar score below 7 at first minute	34/116 (29.3%)	46/125 (36.8%)	80/241 (33.2%)	0.217
Apgar score at fifth minute	9.1 (1.4)	8.8 (1.9)	8.9 (1.7)	0.236
Apgar score below 7 at fifth minute	11/114 (9.6%)	15/123 (12.2%)	26/237 (10.9%)	0.516
Apgar score at tenth minute	9.3 (1.0)	9.1 (1.7)	9.2 (1.4)	0.187
Apgar score below 7 at tenth minute	6/86 (7.0%)	7/84 (8.3%)	13/170 (7.7%)	0.739
Arterial blood cord pH	7.3 (0.1)	7.3 (0.1)	7.3 (0.1)	0.326
Venous blood cord pH	7.4 (0.1)	7.3 (0.1)	7.4 (0.1)	0.047
Composite adverse pregnancy outcome	102/134 (76.1%)	86/144 (59.7%)	188/278 (67.6%)	0.004
Neonatal composite	58/134 (43.3%)	56/144 (38.9%)	114/278 (41.0%)	0.457
Neonatal admission	69/114 (60.5%)	52/123 (42.3%)	121/237 (51.1%)	0.005
NICU admission	48/116 (41.4%)	49/120 (40.8%)	97/236 (41.1%)	0.932
Ventilation	52/117 (44.4%)	50/112 (44.6%)	102/229 (44.5%)	0.976
IMV	27/114 (23.7%)	32/111 (28.8%)	59/225 (26.2%)	0.380
Time requiring IMV, days	3.8 (10.6)	1.9 (4.6)	3.0 (8.6)	0.196
Intubation	18/114 (15.7%)	28/112 (25.0%)	46/226 (20.3%)	0.080
Time requiring intubation, days	12.8 (13.3)	4.8 (5.1)	7.8 (9.8)	0.018
CPAP	32/113 (28.3%)	36/110 (32.7%)	68/223 (30.5%)	0.475
Time requiring CPAP, days	2.9 (6.5)	2.4 (5.1)	2.7 (5.9)	0.640
RDS	32/114 (28.1%)	28/108 (25.9%)	60/222 (27.0%)	0.719
Phototherapy	66/115 (57.4%)	55/109 (50.5%)	121/224 (54.0%)	0.298
Time requiring Phototherapy, days	1.8 (3.4)	1.6 (1.7)	1.7 (2.8)	0.669
IVH	11/116 (9.5%)	7/111 (6.3%)	18/228 (7.9%)	0.376
Grade 1	7/11 (63.6%)	4/7 (57.1%)	11/18 (61.1%)	0.379
Grade 2	2/11 (18.2%)	2/7 (28.6%)	4/18 (22.2%)	
Grade 3	0 (0.0%)	1/7 (14.3%)	1/18 (5.6%)	
Grade 4	2/11 (18.2%)	0 (0.0%)	2/18 (11.1%)	
Neonatal blood transfusion	12/112 (10.7%)	5/98 (5.1%)	17/210 (8.1%)	0.131
Necrotizing Enterocolitis	5/116 (4.3%)	5/111 (4.5%)	10/227 (4.4%)	0.966
Retinopathy	5/113 (4.4%)	4/108 (3.7%)	9/221 (4.1%)	0.786

Continuous variables are summarized as mean (standard deviation) and categorical variables as count (percentage)

Denominators (n/N) are reported for each variable; differences are due to missing data

*PPROM* Preterm prelabor rupture of membranes, *NICU* Neonatal Intensive Care Unit, *IMV* Invasive Mechanical Ventilation, *CPAP* Continuous Positive Airway Pressure, *RDS* Respiratory Distress Syndrome, *IVH* Intraventricular Hemorrhage

Neonatal survival did not differ significantly between latency groups. Apgar scores and umbilical cord pH values were comparable.

### Latency time-to-event analysis: survival analysis

**Fig. 3:**
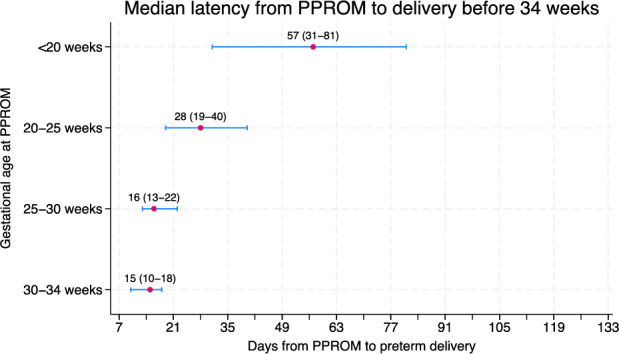
Median latency duration according to gestational age at PPROM

**Fig. 4:**
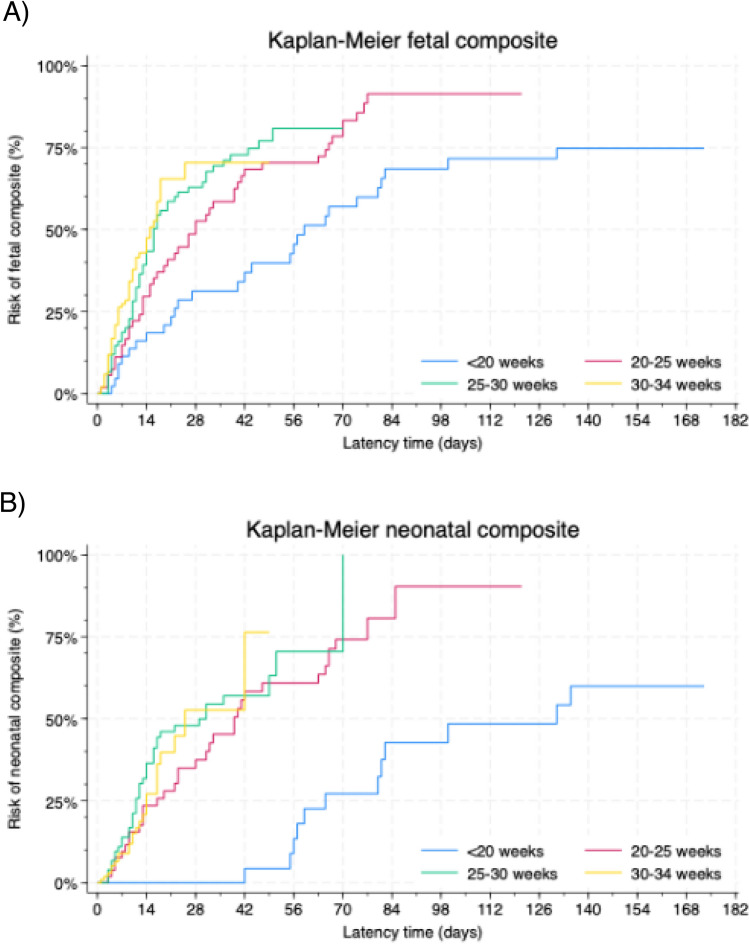
Kaplan–Meier estimates of latency duration according to gestational age at PPROM

Latency time-to-delivery following PPROM was strongly influenced by gestational age at membrane rupture (Spearman’s *ρ* −0.53, *p*<0.001). Median latency decreased progressively with advancing gestational age at PPROM, from 74 days in pregnancies rupturing before 20 weeks to 14 days in those with PPROM between 30 and 34 weeks (Fig. 3). Kaplan–Meier estimates demonstrated a clear inverse relationship between gestational age at PPROM and latency duration (Fig. 4).

**Fig. 5:**
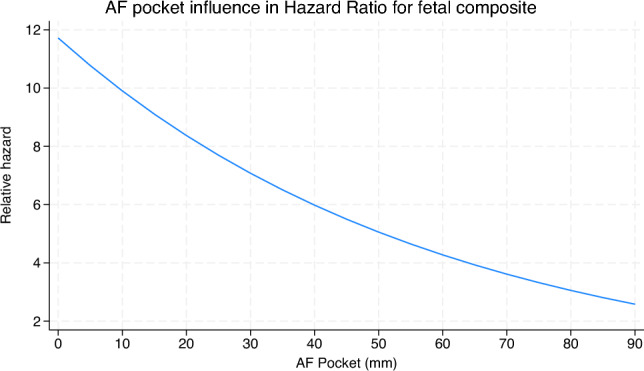
Association between residual amniotic fluid pocket size and the hazard of a composite adverse pregnancy outcome

In Cox proportional hazards models using latency time as the underlying timescale and composite adverse pregnancy outcome as the failure event (176 events, reflecting complete-case analysis for covariates included in the model), gestational age at PPROM was significantly associated with the risk of the composite adverse pregnancy outcome (Table 3). An amniotic fluid pocket >20 mm was independently associated with a lower hazard of delivery. Considering the amniotic fluid pocket as a continuous variable demonstrated a progressive decrease in the hazard of delivery with increasing amniotic fluid pocket size (Fig. 5). Results for the composite outcome were consistent with those observed for its individual components (Supplementary Table S1). Pregnancies with earlier gestational age at PPROM showed a higher risk of stillbirth, whereas later gestational age at rupture was associated with a higher likelihood of medically indicated preterm delivery before 34 weeks. A larger amniotic fluid pocket was associated with a lower risk of both outcomes, although estimates for stillbirth were less precise due to the lower number of events.

**Table 3:** Cox proportional hazards models for composite adverse pregnancy and neonatal outcome

Composite adverse pregnancy outcome	HR	CI 95%	*p*
No. of failures 176			
Gestational age at PPROM, weeks			
<20 weeks	Reference		
20–25 weeks	1.9	1.1–3.1	0.017
25–30 weeks	3.0	1.8–5.1	<0.001
30–34 weeks	4.1	2.3–7.1	<0.001
AF Pocket			
<20 mm	Reference		
>20 mm	0.6	0.4–0.8	0.001
Neonatal Composite	HR	CI 95%	*p*
No. of failures 114			
Gestational age at PPROM, weeks			
<20 weeks	Reference		
20–25 weeks	4.3	2.0–9.1	<0.001
25–30 weeks	7.1	3.2–15.5	<0.001
30–34 weeks	6.7	2.9–15.7	<0.001
AF Pocket			
<20 mm	Reference		
>20 mm	0.7	0.5–1.0	0.085

*HR* Hazard ratio, *CI* Confidence interval, *PPROM* Preterm prelabor rupture of membranes, *AF* Amniotic fluid

When neonatal composite outcomes were analyzed using latency-based time-to-event models, gestational age at PPROM was significantly associated with the occurrence of adverse neonatal events (114 events) (Table 3) in latency-based analyses. In contrast, the amniotic fluid pocket was not independently associated with neonatal composite outcomes.

Sensitivity analyses excluding pregnancies with PPROM before 23 weeks and restricting the cohort to pregnancies before 28 weeks showed consistent results (Supplementary Table S2).

## Discussion

In this retrospective cohort study of pregnancies complicated by PPROM before 34 weeks of gestation, latency duration was strongly influenced by gestational age at membrane rupture and residual amniotic fluid volume. Earlier gestational age at PPROM and larger amniotic fluid pockets were associated with prolonged latency. Gestational age at PPROM was also strongly associated with the occurrence of the composite adverse pregnancy outcome, reflecting the underlying clinical context in which earlier rupture is linked to a higher risk of stillbirth, whereas later gestational ages are more frequently associated with medically indicated delivery before 34 weeks. 

With respect to neonatal outcomes, gestational age at PPROM was significantly associated with adverse neonatal outcomes in latency-based analyses, while residual amniotic fluid volume was not independently associated with neonatal composite outcomes. These findings suggest that the prognostic value of latency in PPROM is largely contextual and closely linked to gestational age rather than latency duration alone.

The inverse relationship between gestational age at PPROM and latency duration observed in our cohort is consistent with established literature [5]. The American College of Obstetricians and Gynecologists reports that birth within 1 week of membrane rupture occurs in at least half of patients with preterm PPROM, with latency inversely correlated with gestational age at membrane rupture. Similarly, the Society for Maternal–Fetal Medicine notes that latency duration in previable and periviable PPROM varies substantially across studies, ranging from 7 days (IQR, 3–29) to 51 days (IQR, 19–107), reflecting differences in gestational age at presentation and patient populations [1, 5]. Our findings of a median latency of 74 days for PPROM before 20 weeks and 14 days for PPROM between 30–34 weeks align with these reported ranges and confirm the strong gestational-age-dependent nature of latency. Seravalli et al. recently reported a median latency of 16 days (IQR 4–27) in a cohort of PPROM before 32 weeks, with variations based on gestational age at membrane rupture, further supporting our observations [2].

The strong association between gestational age at PPROM and latency duration observed in our cohort is consistent with previous reports describing an inverse relationship between gestational age at membrane rupture and the likelihood of prolonged expectant management [3, 6]. Prior studies have consistently shown that pregnancies complicated by earlier PPROM tend to experience longer latency intervals, whereas rupture occurring at more advanced gestational ages is followed by delivery within a shorter timeframe [1, 7, 9].

Our findings align closely with population-based and single-center cohort studies reporting that gestational age at PPROM is the primary factor associated with latency duration, outweighing most baseline maternal or obstetric characteristics[2, 3, 6]. Lorthe et al. and Seravalli et al. demonstrated that latency decreases progressively with advancing gestational age at rupture and that, after accounting for gestational age, latency duration alone is not independently associated with worse neonatal outcomes [2, 3]. By extending these observations to a contemporary cohort managed expectantly before 34 weeks of gestation, our results reinforce the concept that gestational age at PPROM constitutes the dominant contextual determinant of latency duration.

Amniotic fluid volume emerged as an independent factor associated with latency duration in our cohort. Pregnancies with larger amniotic fluid pockets experienced a lower risk of composite adverse pregnancy outcome events (intrauterine death or delivery before 34 weeks) in latency-based Cox models. When modeled as a continuous variable, increasing amniotic fluid pocket was associated with a progressive reduction in the hazard of composite pregnancy adverse outcome consistent with a graded association. Importantly, analysis of the individual components of the composite outcome demonstrated consistent patterns (Table S1). Earlier gestational age at PPROM was associated with a higher risk of stillbirth, whereas later gestational age at rupture was associated with a higher likelihood of medically indicated delivery before 34 weeks. Similarly, a larger amniotic fluid pocket was associated with a lower risk of both outcomes, although estimates for stillbirth were less precise due to the lower number of events. These findings support that the observed associations were not driven solely by the composite definition but reflect underlying clinical processes.

These findings are consistent with previous reports identifying oligohydramnios as a predictor of shortened latency in PPROM and support the concept that amniotic fluid volume reflects the underlying intrauterine environment following membrane rupture [5, 8]. Importantly, however, the amniotic fluid pocket was not independently associated with adverse neonatal composite outcomes in our analyses, indicating that its prognostic relevance may be primarily related to latency dynamics rather than neonatal risk *per se*. Amniotic fluid volume after PPROM is more likely to reflect the underlying intrauterine conditions than to act as a direct causal factor. In clinical practice, residual fluid volume depends on the degree of ongoing fluid loss and fetal urine production, which are influenced by membrane integrity, placental function, and possible intrauterine infection. Therefore, larger amniotic fluid pockets may indicate a more favorable intrauterine environment, whereas oligohydramnios is often associated with more severe clinical compromise. These findings support interpreting amniotic fluid volume as a clinical marker rather than an independent determinant of neonatal outcome [16, 17].

In survival analyses, gestational age at PPROM was significantly associated with adverse neonatal composite outcomes, whereas latency duration did not appear to be independently associated with neonatal outcomes after accounting for gestational age variables. When gestational age at delivery was considered, it showed a strong association with neonatal outcomes, while the association with gestational age at PPROM was attenuated, highlighting the central role of gestational age at delivery in determining neonatal risk. This finding suggests that the prognostic relevance of latency in PPROM may be largely driven by its relationship with gestational age rather than by latency duration. Gestational age at delivery is closely linked to both latency and neonatal outcomes and should be considered when interpreting these associations. These results are consistent with prior studies reporting that, once gestational age at delivery is considered, prolonged latency does not appear to confer additional neonatal risk [4]. Lorthe et al. demonstrated that latency duration did not show an independent association with neonatal survival or early-onset sepsis after adjustment for gestational age at birth [3]. Similar observations have been reported in single-center cohorts, supporting the notion that gestational age remains the dominant contextual factor underlying neonatal outcomes in PPROM [9, 18]. Latency should, therefore, be interpreted within the clinical context of gestational age at rupture, rather than as an independent determinant of outcome. Sensitivity analyses excluding PPROM before 23 weeks and restricting the cohort to <28 weeks showed consistent results. In these subgroups, gestational age remained the main determinant of neonatal outcomes, while amniotic fluid volume was primarily related to latency.

While prolongation of pregnancy remains a central objective in the expectant management of PPROM, extended latency is not without maternal risk. Infectious morbidity, including clinical chorioamnionitis and postpartum infection, as well as placental abruption, has been consistently reported as potential complications of prolonged latency, particularly at earlier gestational ages [1, 10, 11, 19, 20]. As a result, the decision to pursue expectant management requires careful and ongoing reassessment of maternal and fetal status [1, 5].

In this context, our findings underscore the importance of interpreting latency duration within a broader clinical framework. The absence of an independent association between latency duration and adverse neonatal outcomes suggests that the potential neonatal benefit of prolonged latency is primarily mediated through gestational age at delivery rather than latency itself [6, 8, 11, 21]. Accordingly, individualized management strategies that balance the potential gains of gestational age prolongation against maternal risk—rather than targeting latency duration as an isolated goal—may be most appropriate in pregnancies complicated by PPROM [1, 5].

However, conflicting evidence exists. Drassinower et al. reported that PPROM for ≥3 weeks was an independent risk factor for motor (adjusted OR 2.12) and mental (adjusted OR 1.83) Bayley scores of less than 70 at 2 years of age, even after adjusting for gestational age at delivery, neonatal sepsis, and other confounders [18]. This suggests that whereas delivery at later gestational age is associated with improved prognosis for many outcomes, prolonged exposure to an intrauterine environment of PPROM may be an independent risk factor for adverse neurodevelopmental outcomes in some populations. Kieffer et al. similarly reported that very preterm infants born after rupture of membranes before viability had significant neurodevelopmental impairment at 2 years, although outcomes were primarily driven by gestational age at delivery [22].

Conversely, a 10-year follow-up of the PPROMEXIL trials by Simons et al. found that in children born after late preterm PPROM (34–37 weeks), expectant management did not improve long-term outcomes at 10–12 years when compared with induction of labor, with no significant differences in cognition, motor function, or behavior [23]. More recently, Tougas et al. evaluated 5-year neurodevelopmental outcomes in preterm infants born at less than 27 weeks following PPROM and found that while these infants had increased rates of neurodevelopmental impairment, outcomes were primarily related to gestational age at birth rather than latency duration [24].

This finding supports recent evidence suggesting potential benefits of extended expectant management in selected cases. Ghosh et al. demonstrated in a randomized controlled trial of PPROM between 32 and 34 weeks that extended expectant management (delivery at 36 weeks) compared with traditional management (delivery at 34 weeks) resulted in significantly higher mean birth weight, lower NICU stay duration, reduced need for mechanical ventilation, and decreased complications, such as neonatal jaundice and necrotizing enterocolitis, without significant increase in chorioamnionitis or other maternal morbidity [12].

The strengths of this study include its relatively large sample size, inclusion of a wide gestational age range, and the use of latency-based time-to-event analyses to evaluate perinatal outcomes following PPROM. The application of Cox proportional hazards models allowed assessment of the independent associations of gestational age at PPROM and residual amniotic fluid volume with pregnancy adverse and neonatal composite outcomes while appropriately accounting for the timing of events.

Several limitations should be acknowledged. The retrospective and single-center design may limit generalizability and introduce the potential for residual confounding and selection bias. Very early PPROM cases may have been selectively terminated, potentially introducing selection bias; however, most pregnancies in this subgroup continued with expectant management. Histological chorioamnionitis, microbial invasion of the amniotic cavity, and inflammatory biomarkers were not systematically assessed, and long-term neonatal neurodevelopmental outcomes were not evaluated.

The composite adverse pregnancy outcome included medically indicated delivery before 34 weeks, which reflects clinical decision-making and may be influenced by gestational age at rupture. Therefore, it should be interpreted as a pregnancy status outcome rather than a purely fetal endpoint. Neonatal outcomes occur after delivery and should, therefore, be interpreted in relation to latency rather than as true time-to-event endpoints. In our cohort, most neonatal complications occurred within the first 24–48 h after birth, and neonatal deaths were either immediate or preceded by severe early complications, which likely limited the impact of this temporal separation. Finally, limited event numbers in some gestational age subgroups may have reduced statistical power to detect smaller but clinically meaningful differences.

Future prospective, multicenter studies incorporating standardized management protocols, systematic assessment of inflammatory and placental biomarkers, and longer neonatal follow-up are needed to further clarify the complex interplay between latency duration, gestational age, and perinatal outcomes in pregnancies complicated by PPROM.

## Conclusions

In pregnancies complicated by PPROM before 34 weeks of gestation, gestational age at membrane rupture emerged as the main determinant of latency duration and perinatal outcomes. Earlier PPROM was associated with longer latency, whereas latency duration did not appear to provide additional prognostic information for neonatal outcomes after accounting for gestational age. Amniotic fluid volume was associated with prolonged latency and lower risk of intrauterine death but was not clearly associated with neonatal outcomes. These findings support the interpretation of latency as a gestational-age-dependent phenomenon and highlight the importance of individualized expectant management that prioritizes gestational age advancement while balancing maternal risk.

## Supplementary Information

Below is the link to the electronic supplementary material.

Supplementary file1 (DOCX 33 KB)

Supplementary file2 (DOCX 17 KB)

Supplementary file3 (DOCX 19 KB)

## Data Availability

No datasets were generated or analyzed during the current study.

## References

[1] American College of Obstetricians and Gynecologists (2020) Prelabor Rupture of Membranes ACOG Practice Bulletin, Number 217. Obstet Gynecol 135:80–97. 10.1097/AOG.0000000000003700 32080050 10.1097/AOG.0000000000003700

[2] Seravalli V, Colucci C, Di Cencio C et al (2025) Latency to delivery and incidence of adverse obstetric and perinatal outcomes in preterm premature rupture of membranes before 32 weeks. Arch Gynecol Obstet 311:1569–1577. 10.1007/s00404-025-07970-3 39928128 10.1007/s00404-025-07970-3 PMC12055622

[3] Lorthe E, Ancel P-Y, Torchin H et al (2017) Impact of latency duration on the prognosis of preterm infants after preterm premature rupture of membranes at 24 to 32 weeks’ gestation: a national population-based cohort study. J Pediatr 182:47-52.e2. 10.1016/j.jpeds.2016.11.074 28081890 10.1016/j.jpeds.2016.11.074

[4] Bond DM, Middleton P, Levett KM et al (2017) Planned early birth versus expectant management for women with preterm prelabour rupture of membranes prior to 37 weeks’ gestation for improving pregnancy outcome. Cochrane Database Syst Rev. 10.1002/14651858.CD004735.pub4 28257562 10.1002/14651858.CD004735.pub4 PMC6464692

[5] Battarbee AN, Osmundson SS, McCarthy AM, Louis JM (2024) Society for Maternal-Fetal Medicine Consult Series #71: management of previable and periviable preterm prelabor rupture of membranes. Am J Obstet Gynecol 231:B2–B15. 10.1016/j.ajog.2024.07.016 39025459 10.1016/j.ajog.2024.07.016

[6] Müller H, Stähling A-C, Bruns N et al (2022) Latency duration of preterm premature rupture of membranes and neonatal outcome: a retrospective single-center experience. Eur J Pediatr 181:801–811. 10.1007/s00431-021-04245-2 34605998 10.1007/s00431-021-04245-2 PMC8821059

[7] Zhou S, Yang Y, Zhang X et al (2021) Perinatal outcomes of twin pregnancies with preterm premature rupture of the membranes at 24–34 weeks’ gestation. Sci Rep 11:23419. 10.1038/s41598-021-02884-x 34862450 10.1038/s41598-021-02884-x PMC8642529

[8] Ekin A, Gezer C, Taner CE et al (2014) Risk factors and perinatal outcomes associated with latency in preterm premature rupture of membranes between 24 and 34 weeks of gestation. Arch Gynecol Obstet 290:449–455. 10.1007/s00404-014-3227-3 24695905 10.1007/s00404-014-3227-3

[9] Zhou S, Mei L, Zhou W et al (2022) Clinical factors and perinatal outcomes associated with short latency period in twin pregnancies with preterm premature rupture of membranes before 34 weeks: a retrospective study. Front Med 9:839240. 10.3389/fmed.2022.839240 10.3389/fmed.2022.839240 PMC8931478 35308543

[10] Galletta MAK, Schultz R, Sartorelli MFGDOP et al (2023) Clinical characteristics, complications, and predictive model of histological chorioamnionitis in women with preterm premature rupture of membranes. PLoS ONE 18:e0283974. 10.1371/journal.pone.0283974 37023210 10.1371/journal.pone.0283974 PMC10079121

[11] Ronzoni S, Cobo T, D’Souza R et al (2022) Individualized treatment of preterm premature rupture of membranes to prolong the latency period, reduce the rate of preterm birth, and improve neonatal outcomes. Am J Obstet Gynecol 227:296.e1-296.e18. 10.1016/j.ajog.2022.02.037 35257664 10.1016/j.ajog.2022.02.037

[12] Ghosh D, Jena P, Sahu PS et al (2025) Safety and efficacy of extended expectant management in preterm premature rupture of membrane between 32 and 34 weeks of pregnancy-a randomization control trial. Eur J Obstet Gynecol Reprod Biol 310:113971. 10.1016/j.ejogrb.2025.113971 40250040 10.1016/j.ejogrb.2025.113971

[13] International Society of Ultrasound in Obstetrics and Gynecology, Bilardo CM, Chaoui R et al (2023) ISUOG practice guidelines (updated): performance of 11–14‐week ultrasound scan. Ultrasound Obstet Gynecol 61:127–143. 10.1002/uog.26106 36594739 10.1002/uog.26106

[14] NICE Clinical Guideline CG55 (2007) Intrapartum care: care of women and their babies during childbirth. RCOG Press, London 21250397

[15] SEGO (2012) Rotura prematura de membranas. Prog Obstet Ginecol 55:520–540. 10.1016/j.pog.2012.10.002

[16] Weiner E, Barrett J, Zaltz A et al (2019) Amniotic fluid volume at presentation with early preterm prelabor rupture of membranes and association with severe neonatal respiratory morbidity. Ultrasound Obstet Gynecol 54:767–773. 10.1002/uog.20257 30834608 10.1002/uog.20257

[17] Ekin A, Gezer C, Taner CE, Ozeren M (2015) Perinatal outcomes in pregnancies with oligohydramnios after preterm premature rupture of membranes. J Matern Fetal Neonatal Med 28:1918–1922. 10.3109/14767058.2014.972927 25283853 10.3109/14767058.2014.972927

[18] Drassinower D, Friedman AM, Običan SG et al (2016) Prolonged latency of preterm prelabour rupture of membranes and neurodevelopmental outcomes: a secondary analysis. BJOG 123:1629–1635. 10.1111/1471-0528.14133 27245741 10.1111/1471-0528.14133

[19] Baser E, Aydogan Kirmizi D, Ulubas Isik D et al (2020) The effects of latency period in PPROM cases managed expectantly. J Matern Fetal Neonatal Med 33:2274–2283. 10.1080/14767058.2020.1731465 32089027 10.1080/14767058.2020.1731465

[20] Phillips A, Pagan M, Smith A et al (2023) Management and interventions in previable and periviable preterm premature rupture of membranes: a review. Obstet Gynecol Surv 78:682–689. 10.1097/OGX.0000000000001198 38134338 10.1097/OGX.0000000000001198

[21] Younge NE, Saha S, Brumbaugh JE et al (2025) Outcomes of extremely preterm infants exposed to prolonged prelabor rupture of membranes before 24 weeks of gestation. Am J Obstet Gynecol 233:131.e1-131.e14. 10.1016/j.ajog.2025.01.010 39800181 10.1016/j.ajog.2025.01.010 PMC12241454

[22] Kieffer A, Pinto Cardoso G, Thill C et al (2016) Outcome at two years of very preterm infants born after rupture of membranes before viability. PLoS ONE 11:e0166130. 10.1371/journal.pone.0166130 27829004 10.1371/journal.pone.0166130 PMC5102432

[23] Simons NE, de Ruigh AA, van ’t Hooft J et al (2023) Childhood outcomes after induction of labor or expectant management for preterm prelabor rupture of membranes: a 10-year follow-up of the PPROMEXIL trials. Am J Obstet Gynecol 228:588.e1-588.e13. 10.1016/j.ajog.2023.02.007 36787813 10.1016/j.ajog.2023.02.007

[24] Tougas S, Bhullar H, Stritzke A et al (2025) Long-term neurodevelopmental outcomes at 5 years in preterm infants born at <27 weeks’ gestational age following preterm premature rupture of membranes. Am J Perinatol. 10.1055/a-2698-0900 40983094 10.1055/a-2698-0900

