# Supplementary material for: Latency following preterm prelabor rupture of membranes before 34 weeks of gestation and its association with perinatal outcomes: a retrospective cohort study
Source: Arch Gynecol Obstet. 2026 May 16;313(1):220. doi: 10.1007/s00404-026-08463-7 (PMC13346162; doi:10.1007/s00404-026-08463-7)
Supplement: Supplementary file 2 — Supplementary file2 (DOCX 17 KB) [file 404_2026_8463_MOESM2_ESM.docx]

**Table S1** – Multivariable Cox regression analysis for the individual components of the composite adverse pregnancy outcome.

|  | Stillbirth | | | Indicated PTB <34 weeks | | |
| --- | --- | --- | --- | --- | --- | --- |
|  | HR | 95%CI | *p* | HR | 95%CI | *p* |
| **Gestational age at PPROM** |  |  |  |  |  |  |
| < 20 weeks | Reference |  |  | Reference |  |  |
| 20 - 25 weeks | 0.63 | 0.27-1.47 | 0.281 | 3.35 | 1.68-6.69 | **0.001** |
| 25 - 30 weeks | 0.22 | 0.06-0.78 | **0.020** | 7.33 | 3.59-14.96 | **<0.001** |
| 30 - 34 weeks | Not estimable |  |  | 10.62 | 5.08-22.22 | **<0.001** |
| **Amniotic fluid pocket at PPROM** |  |  |  |  |  |  |
| <20 mm | Reference |  |  | Reference |  |  |
| >20 mm | 0.30 | 0.10-0.88 | **0.028** | 0.65 | 0.46-0.91 | **0.012** |
