# Supplementary material for: Latency following preterm prelabor rupture of membranes before 34 weeks of gestation and its association with perinatal outcomes: a retrospective cohort study
Source: Arch Gynecol Obstet. 2026 May 16;313(1):220. doi: 10.1007/s00404-026-08463-7 (PMC13346162; doi:10.1007/s00404-026-08463-7)
Supplement: Supplementary file 3 — Supplementary file3 (DOCX 19 KB) [file 404_2026_8463_MOESM3_ESM.docx]

**Table S2** – Sensitivity analyses according to gestational age at PPROM

|  | Composite adverse pregnancy outcome | | | Neonatal composite outcome | | |
| --- | --- | --- | --- | --- | --- | --- |
|  | HR | 95% CI | *p* | HR | 95% CI | *p* |
| **Gestational age at PPROM** |  |  |  |  |  |  |
| 23–25 weeks | Reference |  |  | Reference |  |  |
| 25–30 weeks | 1.47 | 0.86–2.51 | 0.154 | 1.40 | 0.78–2.51 | 0.260 |
| 30–34 weeks | 1.71 | 0.97–3.02 | 0.064 | 1.11 | 0.57–2.19 | 0.753 |
| **Amniotic fluid pocket at PPROM** |  |  |  |  |  |  |
| ≤20 mm | Reference |  |  | Reference |  |  |
| >20 mm | 0.61 | 0.43–0.88 | **0.007** | 0.64 | 0.42–0.99 | **0.044** |

Panel A. Excluding pregnancies with PPROM before 23 weeks.

|  | Composite adverse pregnancy outcome | | | Neonatal composite outcome | | |
| --- | --- | --- | --- | --- | --- | --- |
|  | HR | 95% CI | *p* | HR | 95% CI | *p* |
| **Gestational age at PPROM** |  |  |  |  |  |  |
| 23–25 weeks | Reference |  |  | Reference |  |  |
| 25–28 weeks | 1.23 | 0.69–2.20 | 0.477 | 1.29 | 0.70–2.39 | 0.417 |
| **Amniotic fluid pocket at PPROM** |  |  |  |  |  |  |
| ≤20 mm | Reference |  |  | Reference |  |  |
| >20 mm | 0.84 | 0.47–1.49 | 0.549 | 0.91 | 0.50–1.65 | 0.756 |

Panel B. Restricted to pregnancies with PPROM before 28 weeks.
